# Computer-Aided Nodule Assessment and Risk Yield (CANARY) may facilitate non-invasive prediction of EGFR mutation status in lung adenocarcinomas

**DOI:** 10.1038/s41598-017-17659-6

**Published:** 2017-12-15

**Authors:** Ryan Clay, Benjamin R. Kipp, Sarah Jenkins, Ron A. Karwoski, Fabien Maldonado, Srinivasan Rajagopalan, Jesse S. Voss, Brian J. Bartholmai, Marie Christine Aubry, Tobias Peikert

**Affiliations:** 1 Pulmonary & Critical Care Medicine, Mayo Clinic, Rochester, MN USA; 2 Laboratory Medicine & Pathology, Mayo Clinic, Rochester, MN USA; 3 Biomedical Statistics & Informatics, Mayo Clinic, Rochester, MN USA; 4 Biomedical Imaging Resource, Mayo Clinic, Rochester, MN USA; 5 Allergy, Pulmonary & Critical Care Medicine, Vanderbilt University, Nashville, TN USA; 6 Thoracic Radiology, Mayo Clinic, Rochester, MN USA

## Abstract

Computer-Aided Nodule Assessment and Risk Yield (CANARY) is quantitative imaging analysis software that predicts the histopathological classification and post-treatment disease-free survival of patients with adenocarcinoma of the lung. CANARY characterizes nodules by the distribution of nine color-coded texture-based exemplars. We hypothesize that quantitative computed tomography (CT) analysis of the tumor and tumor-free surrounding lung facilitates non-invasive identification of clinically-relevant mutations in lung adenocarcinoma. Comprehensive analysis of targetable mutations (50-gene-panel) and CANARY analysis of the preoperative (≤3 months) high resolution CT (HRCT) was performed for 118 pulmonary nodules of the adenocarcinoma spectrum surgically resected between 2006–2010. Logistic regression with stepwise variable selection was used to determine predictors of mutations. We identified 140 mutations in 106 of 118 nodules. TP53 (n = 48), KRAS (n = 47) and EGFR (n = 15) were the most prevalent. The combination of Y (Yellow) and G (Green) exemplars, fibrosis within the surrounding lung and smoking status were the best discriminators for an EGFR mutation (AUC 0.77 and 0.87, respectively). None of the EGFR mutants expressing TP53 (n = 5) had a good prognosis based on CANARY features. No quantitative features were significantly associated with KRAS mutations. Our exploratory analysis indicates that quantitative CT analysis of a nodule and surrounding lung may noninvasively predict the presence of EGFR mutations in pulmonary nodules of the adenocarcinoma spectrum.

## Introduction

Lung cancer remains the leading cancer-related cause of death in the United States, and continues to have a poor prognosis with a 5-year survival rate of 18%^1^. As the United States Preventative Services Task Force recommendations to screen high-risk individuals for lung cancer are implemented^2^, we will see increased identification of early-stage lung cancer. The majority of these lesions are part of the lung adenocarcinoma spectrum^3^.

Adenocarcinomas frequently harbor driver mutations such as Kirsten rat sarcoma (KRAS) and epithelial growth factor receptor (EGFR) mutations. The clinical significance of these mutations is based on favorable therapeutic responses of EFGR-mutated tumors to EGFR tyrosine kinase inhibitor (TKI) therapy yielding improved survival with decreased toxicity compared to standard platinum-based cytotoxic chemotherapy^4,5^. Conversely, harboring a KRAS mutation predicts a poorer response to EGFR-targeted TKIs^6^. Invasive tissue sampling is required to investigate the presence of a targetable mutation.

While EGFR positivity is more prevalent among women and never smokers^7–14^, KRAS is more prevalent in smokers and former smokers. KRAS and EGFR mutations tend to be mutually exclusive. It is also unclear how these driver mutations affect the natural history of non-small cell lung cancer (NSCLC)^15–17^. Prior research suggests that lung adenocarcinomas harboring EGFR mutations are characterized by common radiologic features, such as an increased amount of ground glass opacity (GGO) however, published data have been inconsistent, and no definitive radiological pattern has emerged^7–14,18^. There is limited data regarding the radiologic features of KRAS-positive tumors – though an association exists between spiculation of the nodule and KRAS positivity^18,19^. Tumors interact with the tumor-free surrounding lung tissue in multiple ways. Field effects within the lung tissue may predispose to the development of the tumor. However, tumor growth pattern, stromal reactions and the effects cytokine-mediated changes on surrounding tissue^20^ may alter the radiological characteristics of the lung tissue around the tumor. We hypothesize that these changes vary in the presence of different driver mutations, and that these differences can be detected by quantitative CT analysis.

Radiomics refers to reproducible quantitative CT analytic features that correlate with tumor biology and behavior^21^. A radiomics-based approach allows non-invasive comprehensive volumetric characterization of the tumor and surrounding lung tissue. Compared to tissue biopsy, it is more resilient to sampling error and tumor heterogeneity and may reflect molecular changes within the tumor including driver mutations^22^.

The Computer-Aided Nodule Assessment and Risk Yield (CANARY) tool comprehensively analyses, voxel-by-voxel, the distributions of 9 texture-based exemplars within a nodule, as previously described^23^. The exemplars are color coded as Violet (V), Indigo (I), Blue (B), Green (G), Yellow (Y), Orange (O), Red (R), Cyan (C), and Pink (P). Volumetric distributions of each exemplar are summarized in a glyph displaying the proportional makeup of the nodule. Histopathologic evaluation of adenocarcinoma for features such as lepidic growth (tumor growth along pre-existing alveolar structures) predicts improved disease free survival (DFS) after tumor resection with the best survival in adenocarcinoma *in-situ* (100% lepidic growth) compared with minimally-invasive adenocarcinoma (MIA) and invasive adenocarcinoma (IA)^24,25^. The distribution of the CANARY exemplars correlates well with consensus histopathology with B-C-G corresponding to lepidic growth (visually more ‘ground glass’ density) and V-I-R-O (generally more solid density) correlating with the invasive component of the tumor. Furthermore, natural clustering of these glyphs facilitates the risk stratification of lung adenocarcinomas into good (G), intermediate (I) and poor (P) survival groups independent of stage^23,25,26^.

CANARY may add synergistic information regarding prognosis when paired with mutational analyses – and furthermore may be able to detect imaging signatures of common driver mutations, thus eliminating or reducing the need for further invasive testing to guide individualized therapy for lung cancer patients. Next-generation sequencing refers to multi-gene targeted massive parallel sequencing. Mayo Clinic Laboratories has clinically implemented a 50-gene panel that can be performed using as little as 10 ng of DNA using the Ampliseq Cancer Hotspot Panel v2 (Thermo Fischer Scientific) to amplify tumor DNA. This panel targets over 2800 possible somatic mutations within 50 cancer-associated genes facilitating individualized cancer management. Given that KRAS and EGFR are among the most clinically-relevant driver mutations, we selected these two mutations to look for CANARY signatures that could non-invasively identify these mutations.

We hypothesized that the V-I-R-O pattern will be seen more frequently in KRAS-positive tumors while B-C-G will be seen more frequently in EGFR-positive tumors. Additionally we performed quantitative textural analysis of the tumor-free surrounding lung parenchyma using Computer Aided Lung Informatics for Pathology Evaluation and Rating (CALIPER) to determine whether loco-regional lung parenchymal changes, particularly the presence of low attenuation areas and fibrosis, are predictive of EGFR or KRAS^27^.

## Material and Methods

### Subject selection

In a previously-analyzed retrospective cohort of 264 clinical stage I cases of resected adenocarcinoma of the lung between January 2006 and 2009^25^ we identified 129 adequate histopathological specimens in the Mayo Clinic tissue registry with a non-contrast preoperative high resolution computed tomography (HRCT) scan of the chest (≤3 months prior to resection). Clinical data including disease free survival (DFS) were collected from the Mayo Clinic electronic medical records. The study was approved by the Mayo Clinic Institutional Review Board for an informed consent waiver (protocol 14–000666). All subjects reviewed had previously consented to participate in retrospective research. All research was performed in accordance with relevant guidelines and regulations. Archival formalin-fixed, paraffin embedded (FFPE) tissue was available for all cases analyzed. Final analysis was performed on one nodule per subject.

### Nodule analysis and CANARY development

The development of CANARY has been previously described^23^. Briefly, an experienced thoracic radiologist (BJB) arbitrarily selected 774 regions of interest (ROI, 9 × 9 voxels) along the spectrum of histologically-proven lung adenocarcinomas from 37 randomly-selected tumors. The similarities between the ROIs were compared and clustered using pairwise similarity metric and affinity propagation clustering^28^.

**Figure 1:**
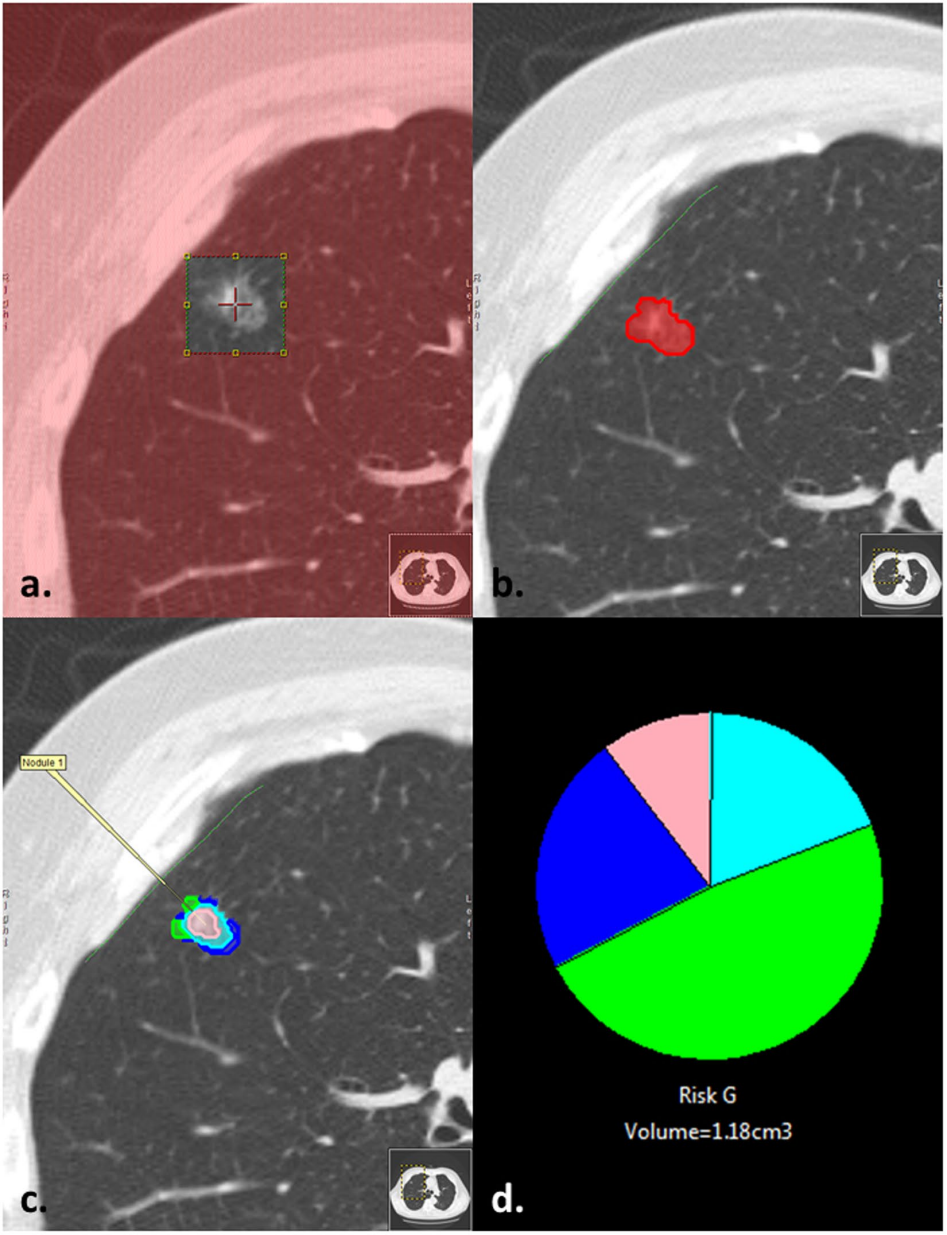
(**a**–**d**) Example of nodule characterization by CANARY in which the (**a**) user selects a seed in the center of the nodule guided by x, y and z axis, (**b**) a mask is generated encompassing the nodule’s volume in which CANARY analysis (**c**) assigns each voxel the color code of the closest exemplar which is also represented by a glyph (**d**) displaying the relative proportion of each exemplar within a nodule.

The location of all surgically-resected nodules was known a priori. Each selected nodule was extracted with a supervised approach using constrained region seed growing. Region growing was restricted to ground glass or reticular voxels connected to the seed voxel. After an initial mask was applied to the nodule, each nodule underwent editing, if required, by the user to ensure the nodule volume was captured in its entirety. Each voxel is analyzed and assigned the color code of the nearest exemplar (Fig. 1). Based on the distribution of the exemplar within a given nodule all nodules are assigned to one of the three “risk” groups correlating with post-resection DFS^23,25,26^. Most (89%) scans were volumetric noncontrast HRCT (less than 3 mm contiguous slices). The remaining scans were 3.75–5 mm contiguous slices. 95% had no edge-enhancing and 5% underwent a smoothing algorithm (3 × 3 median filtering) to remove the kernel edge artifact to allow processing by CANARY. Data in submission (Nakajima, 2017) demonstrated excellent inter-user reproducibility of CANARY and data presented in abstract (Clay, 2017, World Congress on Thoracic Imaging) showed excellent repeatability of CANARY analysis across different acquisition techniques, slice thickness and reconstruction kernels.

### Tumor-free surrounding lung analysis

**Figure 2:**
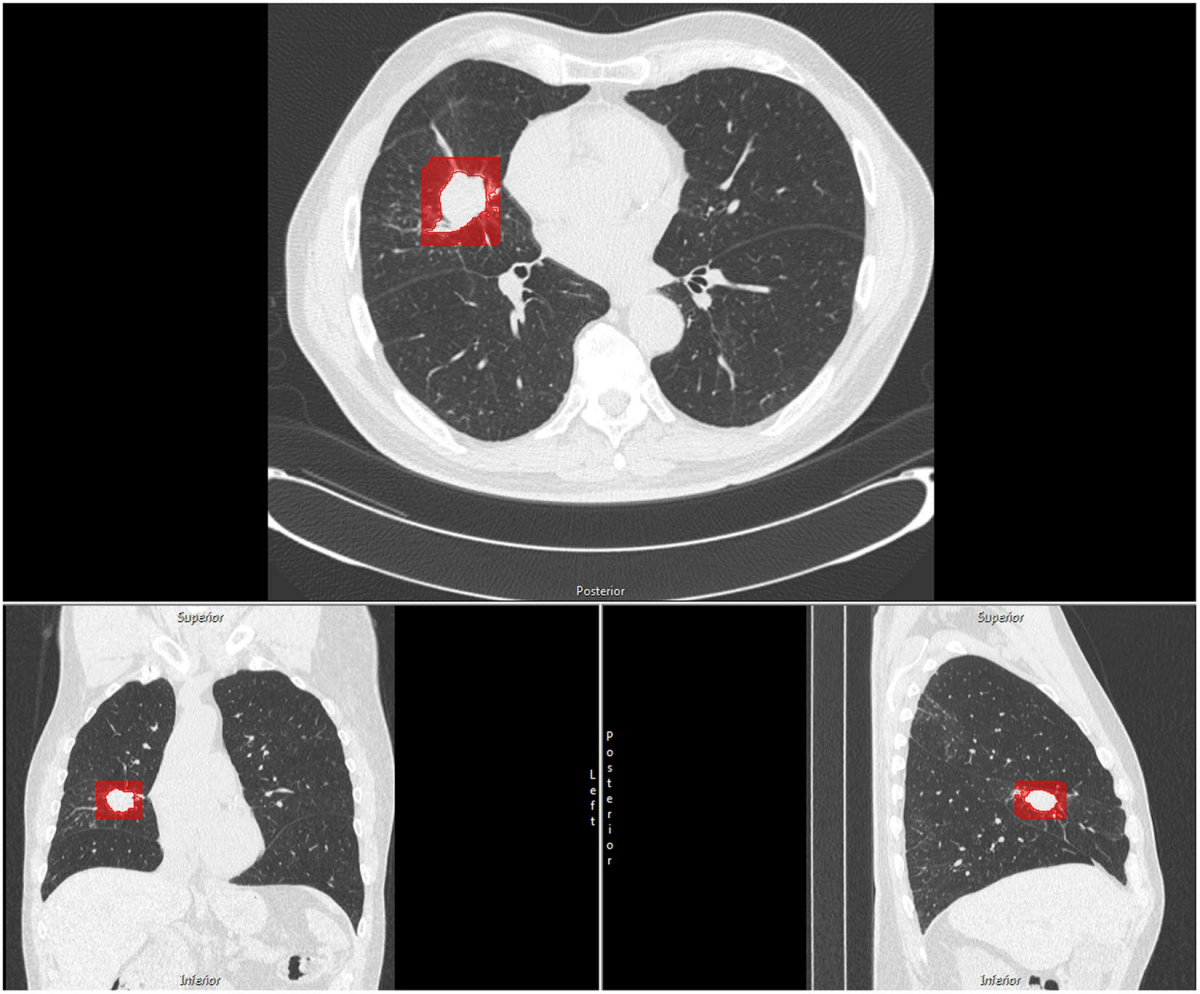
Representation in red of the tumor-free surrounding lung for an adenocarcinoma in the right middle lobe. The area highlighted in red was analyzed by CALIPER for low attenuation and fibrosis shown in the axial, coronal and sagittal planes. Each nodule underwent analysis of the tumor-free surrounding lung characteristics in this manner.

CALIPER is quantitative CT analysis software that both segments the lung parenchyma classifies it into subtypes (normal (N), low attenuation (LA), ground glass (GG), reticular densities (R) and honeycomb change (HC). CALIPER development and validation is detailed previously – but in brief, radiologist-selected 15 × 15 × 15 voxel volumes of interest (VOI) were allowed to cluster by affinity propagation and paired down to 5 basic clusters. These CALIPER classifications showed strong agreement with radiologist classification, physiologic data and clinical phenotypes^27,29^. CALIPER analysis of a 10 mm surrounding mask of tumor-free lung was performed. These results constituted an additional variable to consider in building a model to predict mutational status (Fig. 2).

### Mutation analysis

DNA extraction was performed on archival FFPE tissue obtained at the time of initial surgery. We then performed targeted polymerase-chain reaction (PCR)-based sequencing with a 50 gene panel of common solid tumor driver mutations. DNA amplification was performed with the Ampliseq Hostspot Panel v2 (Life Technologies) to target common mutations in 50 known cancer-associated genes.

### Statistical analysis

Mutation prevalence was compared with gender, prognostic categories, and smoking status using Fisher’s exact tests or chi-square tests as appropriate. Age was compared with mutation status by Wilcoxon rank sum tests. Logistic regression with stepwise variable selection was used to determine the best exemplar predictors of mutations. Receiver operating characteristic (ROC) analyses were used to identify a cut-off for exemplars to achieve an 80% sensitivity to detect EGFR mutation. Post curative resection disease-free survival (DFS) was illustrated with Kaplan-Meier curves by CANARY prognosis and mutation status. Associations between DFS with prognosis, mutations, and exemplars were assessed with likelihood ratio tests from Cox proportional-hazards regression models. All p-values were two-tailed, and p-values less than 0.05 were considered statistically significant. Analyses were performed using SAS version 9.4 (copyright 2002–2012 by SAS Institute Inc., Cary, NC) and R (2014, R Foundation for Statistical Computing, Vienna, Austria).

## Results

DNA was successfully extracted and analyzed in 118 of the 129 cases. Patient demographics are summarized in Table 1. 106 of the 118 nodules had at least one mutation detected and a total of 140 mutations were identified. 47 tumors harbored the KRAS mutation while 15 tumors harbored the EGFR mutation. These two mutations were mutually exclusive. Of the 15 EGFR mutants, 6 were the L858R point mutation in exon 21 and the rest were exon 19 deletions. Additional identified mutations included TP53 (n = 48), STK11 (n = 11), BRAF (n = 4), ATM (n = 3), PTEN (n = 3), PIK3 (n = 2), SMAD4 (n = 1), MET (n = 1), APC (n = 1), GRAS (n = 1), CDK2NA (n = 1), RB (n = 1), and PTPN11 (n = 1). EGFR mutations were more common among never smokers, while KRAS was more frequently mutated in current and former smokers (p < 0.0001, p = 0.02, respectively). There was no significant gender or age difference between patients with EGFR or KRAS mutations versus the wild type. (Tables 2 and 3) There was no significant difference in median nodule volume between EGFR versus wild type tumors (p = 0.192).

**Table 1:** Patient demographics and pathological stage.

Demographics	*n* = 118
Age at diagnosis: median years (range)	68 (35–91)
Gender *n* (%)	
Women	65 (55%)
Men	53 (45%)
Smoking *n* (%)	
Current	27 (23%)
Former	77 (65%)
Never	14 (12%)
Pathologic TNM stage *n* (%)	
I	94 (80%)
II	12 (10%)
III	11 (9%)
IV	1 (1%)

**Table 2:** Characteristics of EGFR mutants. Nodule volume displayed as median + interquartile range. P values calculated by chi square or Fischer exact for <5 counts and Wilcoxon Rank Sum for continuous variables.

	EGFR positive (*n* = 15)	Wild type (*n* = 103)	p
Gender			0.17
Male	4 (26.7%)	49 (47.6%)	
Female	11 (73.3%)	54 (52.4%)	
Age at diagnosis			0.11
Years: median (range)	73 (42–89)	68 (35–91)	
Smoking status			<0.0001
Never	8 (53.3%)	6 (5.8%)	
Former	7 (46.7%)	70 (68.0%)	
Current	0 (0.0%)	27 (26.2%)	
Nodule volume (cc)	2.9 (1.8–6.0)	2.1 (1.1–3.5)	0.192
CANARY prognosis			0.16
G	2 (13.3%)	9 (8.7%)	
I	12 (80.0%)	65 (63.1%)	
P	1 (6.7%)	29 (28.1%)

**Table 3:** Characteristics of KRAS mutants.

	KRAS positive (*n* = 47)	Wild type (*n* = 71)	p
Gender			0.43
Male	19 (40.4%)	34 (47.9%)	
Female	28 (59.6%)	37 (52.1%)	
Age at diagnosis			0.48
Years: median (range)	68 (46–82)	69 (35–91)	
Smoking status			0.02
Never	1 (2.1%)	13 (18.3%)	
Former	36 (76.6%)	41 (57.7%)	
Current	10 (21.3%)	17 (23.9%)	
Nodule volume (cc)	2.5 (0.2–22.4)	2.0 (0.2–14.5)	0.19
CANARY prognosis			0.06
G	7 (14.9%)	4 (5.6%)	
I	25 (53.2%)	52 (73.2%)	
P	15 (31.9%)	15 (21.1%)	

**Figure 3:**
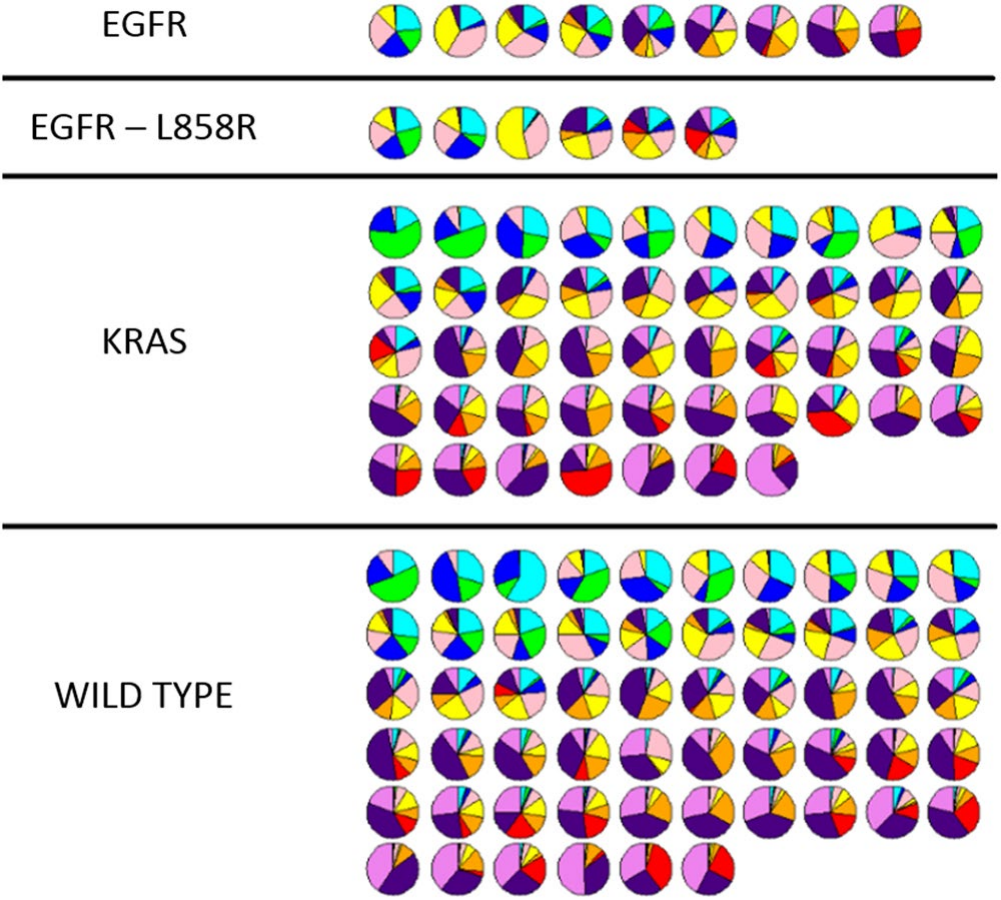
CANARY glyphs representing each unique nodule demonstrate proportionate representation of each CANARY exemplar. Glyphs are arranged by mutation status from left to right in order of parametric signatures correlating with progressively more invasive histopathology. Wild type denotes WT status for both EGFR and KRAS.

**Figure 4:**
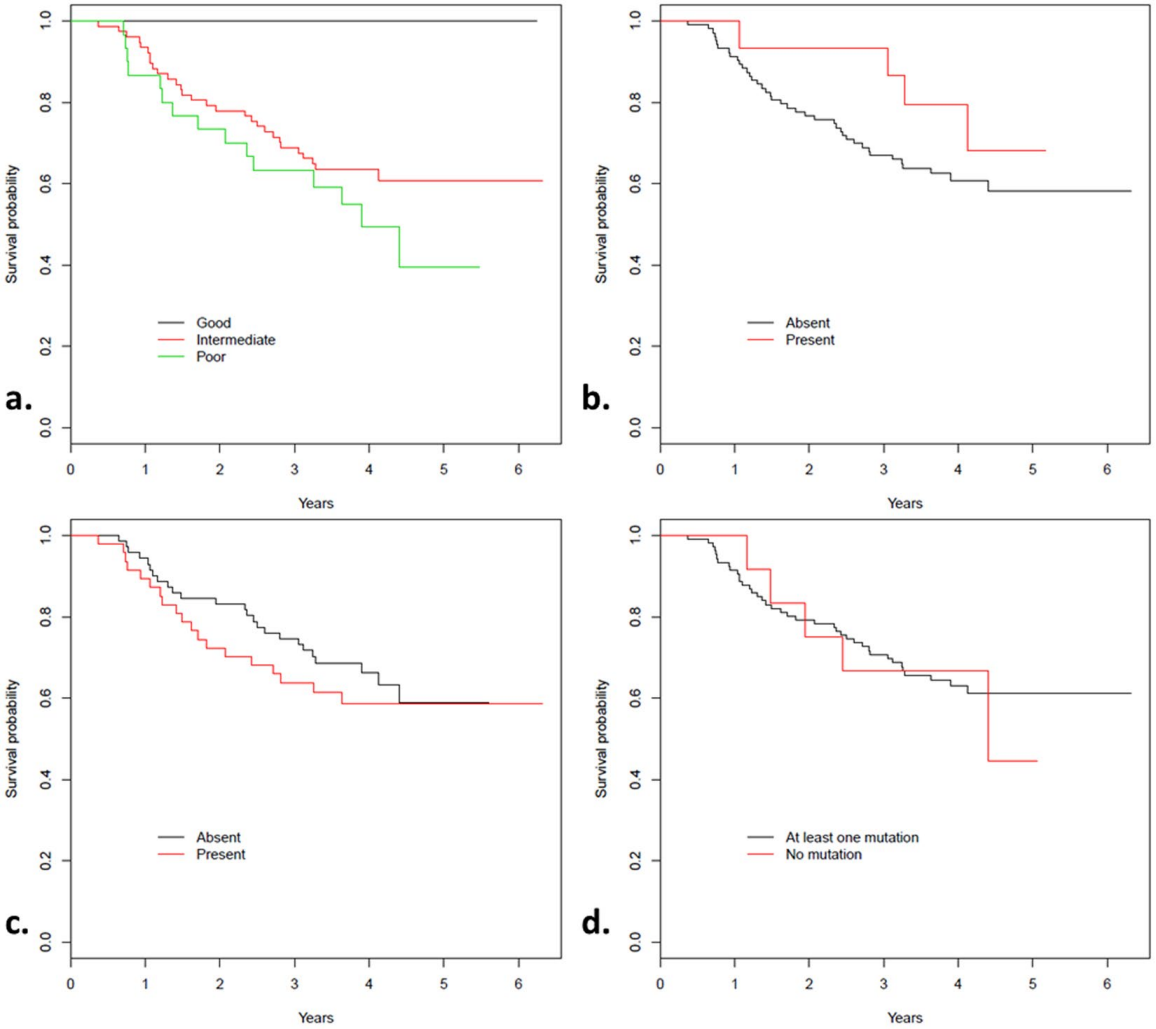
(**a**–**d**) Kaplan Meier survival curves depict likelihood of survival by (**a**) CANARY prognosis (p = 0.002), (**b**) presence of EGFR mutation (p = 0.26), (**c**) presence of KRAS mutation (p = 0.48) or (**d**) presence of any driver mutation (p = 0.78).

CANARY analysis was performed on each nodule generating a representative glyph that shows the proportional distribution of the 9 CANARY exemplars (Fig. 3). This data represents a subset of previously reported data^25^. While CANARY prognostic categories Good (G), intermediate (I) and Poor (P) predicted disease free survival (p = 0.002) independent of stage, there were no statistical DFS differences based on EGFR, KRAS or any detected mutation by our 50 gene panel using Kaplan Meier analysis (p = 0.26, 0.48, 0.78, respectively, Fig. 4). While CANARY prognosis categories did not differ significantly between tumors with EGFR and KRAS mutations (p = 0.16, p = 0.06, respectively), we found that an increase of the V-I-R-O or a decrease of the Y-P component (which is negatively correlated with V-I-R-O correlation = −0.78) within a tumor was associated with a lower likelihood of EGFR positivity (p = 0.01 for V-I-R-O (area under the curve (AUC) = 0.70), p = 0.02 for Y-P (AUC = 0.68)). Each 10% decrease of V-I-R-O component per nodule was associated with a 23% increase in the odds of containing an EGFR mutation (OR = 1.23, 95% CI 1.04–1.46). In contrast the B-C-G exemplars did not significantly affect the odds of harboring an EGFR mutation (p = 0.16). Using receiver operating characteristic (ROC) analysis, we identified that a cut-off for V-I-R-O of ≤ 71% tumor volume identifies EGFR mutations with a sensitivity of 80% and a specificity of 52% (AUC = 0.66). Similarly, since V-I-R-O and Y-P are strongly negatively correlated, we identified a cut-off for Y-P of ≥ 23.5% tumor volume with a sensitivity of 80% and specificity of 53% (AUC = 0.67).

EGFR-positive tumors also had significantly less fibrosis (summed GG + R + HC) and low attenuation areas in the tumor-free surrounding lung, (p = 0.007 and 0.001, respectively).

Using logistic regression and stepwise variable selection to choose among the 9 individual exemplars, none were found to be significant in predicting KRAS positivity. Using the same methods to analyze the relationship between the exemplars and EGFR status we found both Y (p = 0.002) and G (p = 0.008) to be significant and that the odds of harboring an EGFR mutation increase as the percentage of Y and G in a nodule increase. Using recursive partitioning as an additional method to determine the best discriminator of EGFR status, the best predictor was found to be the Y exemplar. In univariate performance, the Y exemplar had an AUC of 0.69. Using a combined Y and G model increased the AUC to 0.77. Adding smoking status to the Y + G model increased the AUC to 0.85. Y retained significance in this model (p = 0.003) whereas G did not (p = 0.06). Y and smoking status remained significant in this model (OR = 1.09, p = 0.05; and OR = 20.0, p = 0.0001, respectively). Neither fibrosis nor the G exemplar were significant in this model (p = 0.09, 0.22, respectively). Fibrosis and low attenuation were not combined in a model due to their high correlation of 0.95 – which would add to model instability. The degree of fibrosis found in the tumor-free surrounding lung was also predictive of EGFR status with an AUC 0.72. A combined model of smoking status, Y + G and CALIPER-determined fibrosis predicted having an EGFR mutation with an AUC of 0.87. Both fibrosis and smoking status retained significance in this model (p = 0.04, p < 0.0001, respectively).

TP53 mutations were detected in 40.7% of our study. None of the TP53 mutant nodules were classified as a good (G) by CANARY (p = 0.002) and all 5 of the 15 EGFR-mutant tumors with concurrent TP53 mutations were classified as intermediate (I) by CANARY.

We performed additional exploratory analysis comparing the CANARY exemplars of tumors with the L858R EGFR point mutation (n = 6) to the other EGFR mutants (n = 9) and wild type cases (n = 103). While the median V-I-R-O component was lower in the L8585R group compared with other EGFR mutants (17.6% versus 49.1%), this difference did not reach statistical significance (p = 0.38). However there was significantly less V-I-R-O component among the L858R mutants than the wild type cases (17.6% versus 73.0%, p = 0.02). Additionally there was no difference in DFS between these groups (p = 0.71).

## Discussion

Our study indicates that CANARY, especially the absence of V-I-R-O and the presence of Y-G exemplars within HRCT-imaged adenocarcinoma of the lung may noninvasively predict the presence of an EGFR mutation. This prediction is strengthened by analysis of the tumor-free surrounding lung. Radiomics features may become valuable adjuncts to patient care especially since these features (CANARY exemplars) have been proven to be more predictive of post-resection DFS when compared with EGFR or KRAS mutation status alone^25,26^. Currently the American College of Pathologists, International Association for the Study of Lung Cancer, the Association for Molecular Pathology and other major organizations recommend the routine testing of targetable molecular abnormalities for all lung adenocarcinomas^30^. This approach requires an invasive tissue biopsy, exposing patients to iatrogenic complications. In addition, molecular testing of invasive tissue samples or resected tumor specimens typically only includes a minute portion of the tumor and is susceptible to sampling error and tumor heterogeneity. Furthermore, small samples may be insufficient to perform these ancillary studies, potentially resulting in the need to re-expose patients to invasive procedures to obtain adequate material^31,32^.

Consistent with prior literature, we found that KRAS correlated with increased tobacco exposure while EGFR correlated with decreased tobacco exposure^33^. This may explain why we saw increased low attenuation lung (emphysema) surrounding the non-EGFR-mutated tumors. Invasion of fibroblasts into the tumor-free surrounding lung driven by carcinogenic cytokines is thought to facilitate tumor growth and invasion^20^. Our finding of increased fibrosis in the tumor-free surrounding lung of wild-type adenocarcinoma, particularly in a tobacco-exposed cohort, may represent the radiologic correlate of this phenomenon. Our study had a low incidence of EGFR mutations – though this may be due to our North American patient population and not specifically enriching our cohort for EGFR mutations. The majority of our subjects were current or former smokers, also lowering the likelihood of harboring an EGFR mutation and increasing the likelihood of KRAS mutations. We did not have any ALK mutations, likely due to our small n and its relative infrequency in NSCLC^10,34^.

Non-invasive comprehensive analysis of the tumor volume using cross-sectional imaging carries a decreased risk of morbidity compared to biopsy and can account for tumor heterogeneity. Other investigators previously demonstrated a number of clinical-radiological characteristics and more recently described radiomic features of the tumor can predict the presence of molecular abnormalities specifically EGFR mutations^7–9,11–14,17,22,35^. These studies, however, are quite heterogeneous and make use of different imaging modalities such as positron-emission tomography – whereas the CANARY exemplars are robust in their reproducibility and have been previously validated among large datasets^25,26^.

EGFR has multiple possible mutations, however the Exon 21 L858R point mutation and Exon 19 deletion account for the majority of EGFR mutations in NSCLC^36^. The L858R EGFR mutation has distinct clinical behavior compared with the 19 Exon deletion, with a lower likelihood of responding to EGFR-targeted therapy and worse DFS^37^. Lee and colleagues recently reported that L858R EGFR mutant cases may have unique radiologic and histopathologic features, specifically increased percentage of GGO within the nodule, and a predominantly lepidic pattern of growth, compared with cases carrying other EGFR mutations^8^. Other studies yielded mixed results regarding the radiologic characteristics of tumors harboring the L858R mutation^7,12^. Although we observed a trend towards less V-I-R-O among the small group (n = 6) of L858R EGFR mutant cases, the difference did not reach statistical significance and additional cases are needed to explore this association. EGFR has diverse mutations and as a whole is not clearly tied to outcome in adenocarcinoma of the lung^38^. We did not find a relationship between BCG and EGFR status as originally hypothesized – but rather the individual exemplars Y and G. BCG is tied to indolent histopathology and good prognosis in adenocarcinoma of the lung^23,25^, so the lack of a relationship between EGFR and BCG makes sense. The histopathologic correlate of Y and G is not entirely clear–a mix of an exemplar associated with good prognosis (G) and associated with intermediate prognosis (Y) – however EGFR does not clearly impact prognosis either^38^. Perhaps this mix of exemplars suggests increased tumor heterogeneity in EGFR-mutated tumors – but this needs further determination.

There was a notable absence of TP53 mutation in any lesions classified as good (G) by CANARY (DFS = 100%). This finding correlates with the known association of TP53 with poor outcomes in lung adenocarcinoma and unfavorable response to EGFR-TKIs^39,40^, and may have influenced the CANARY signature in the 5 EGFR mutant cases harboring concurrent TP53 mutations.

Limitations of our study include its retrospective nature, single center design and the relatively small number of cases. The small number of EGFR mutations and other less common molecular changes may make it difficult to detect differences between mutation subtypes. We are currently planning a larger multicenter study to mitigate these limitations. This will also allow us to evaluate the radiological features of tumors with less common molecular abnormalities such ALK translocations, which have been demonstrated to have less GGO^14^. Though the definitive treatment in early stage lung cancer is resection, applying quantitative CT analysis tools to late stage cancer may facilitate therapy choice without the need for a biopsy, and its application to early stage cancer could open the door to explore additional adjuvant therapy.

## Conclusions

In conclusion, the distribution of density-based CANARY exemplars and quantitative CT analysis of the immediate tumor-free surrounding lung may predict the presence of EGFR mutations in lung adenocarcinomas. As volumetric CT-based CANARY analysis is non-invasive and accounts for tumor heterogeneity, CANARY may prove to be a useful radiologic biomarker beyond its validated role for non-invasive histological assessment and stratification of lung adenocarcinomas. The specified prediction cutoffs found in our trial and whether radiomics features can predict tumor response to molecularly-targeted therapy deserves future study.

### CANARY software availability

CANARY software is currently licensed to Imbio LLC (Minneapolis, MN). The software is available through Imbio by request. In addition the Mayo Clinic has been sharing this software with interested research collaborators by request and we hope to expand this process. Please address requests to the corresponding author.
